# Challenges and perspectives of tendon-derived cell therapy for tendinopathy: from bench to bedside

**DOI:** 10.1186/s13287-022-03113-6

**Published:** 2022-09-02

**Authors:** Ziming Chen, Peilin Chen, Monica Zheng, Junjie Gao, Delin Liu, Allan Wang, Qiujian Zheng, Toby Leys, Andrew Tai, Minghao Zheng

**Affiliations:** 1 Division of Surgery, Centre for Orthopaedic Research, Medical School, The University of Western Australia, Nedlands, WA 6009 Australia; 2 Perron Institute for Neurological and Translational Science, Nedlands, WA 6009 Australia; 3 Department of Orthopaedic Surgery, Sir Charles Gairdner Hospital, Nedlands, WA 6009 Australia; 4 Department of Orthopaedic Surgery, Shanghai Jiao Tong University Affiliated Shanghai Sixth People’s Hospital, Shanghai, 200233 China; 5 The Second School of Clinical Medicine, Southern Medical University, Guangzhou, 510000 Guangdong China; 6 Department of Orthopedics, Guangdong Provincial People’s Hospital, Guangdong Academy of Medical Sciences, Guangzhou, 510000 Guangdong China

**Keywords:** Tendon, Tendon-derived stem cells (TDSCs), Myotendinous junction (MTJ), Midsubstance of tendon, Enthesis, Tendinopathy, Tendon-derived cell therapy

## Abstract

Tendon is composed of dense fibrous connective tissues, connecting muscle at the myotendinous junction (MTJ) to bone at the enthesis and allowing mechanical force to transmit from muscle to bone. Tendon diseases occur at different zones of the tendon, including enthesis, MTJ and midsubstance of the tendon, due to a variety of environmental and genetic factors which consequently result in different frequencies and recovery rates. Self-healing properties of tendons are limited, and cell therapeutic approaches in which injured tendon tissues are renewed by cell replenishment are highly sought after. Homologous use of individual’s tendon-derived cells, predominantly differentiated tenocytes and tendon-derived stem cells, is emerging as a treatment for tendinopathy through achieving minimal cell manipulation for clinical use. This is the first review summarizing the progress of tendon-derived cell therapy in clinical use and its challenges due to the structural complexity of tendons, heterogeneous composition of extracellular cell matrix and cells and unsuitable cell sources. Further to that, novel future perspectives to improve therapeutic effect in tendon-derived cell therapy based on current basic knowledge are discussed.

## Introduction

Tendinopathy is the most frequently occurring musculoskeletal disease, with up to 2% of adults reported to suffer from tendon disorders in their life [1, 2], and up to 50% of musculoskeletal injuries in the USA are tendon-related disorders [3]. The incidence of tendon injury and subsequent tendinopathy is bi-modal, occurring most predominantly in those 25–40 years old and over 60 years old [4, 5]. Acute injuries resulting from high-energy activities are prevalent in younger patients, while older patients experience lower-energy injuries in tendons with pre-existing degeneration. Common symptoms of tendinopathy are pain and swelling at the injured site, followed by subsequent disability [6]. Return to activity time is dependent on the nature and severity of the injury, patient age and rehabilitation undertaken, and can vary between 3 and 9 months [7, 8]. Thus, it is both the symptoms and extended recovery period for tendon injuries that adversely affects an individual’s activities of daily living (ADLs) and poses a significant burden to those individuals and our society.

The current treatment objectives for tendinopathy are to treat pain and restore movement and function to affected joints/limbs. For the less severe cases, conservative management is first-line, and may include rest, physical therapy, rehabilitative exercises, activity modification and non-steroidal anti-inflammatory drugs (NSAIDs) [9]. Occasionally, other non-surgical interventions such as corticosteroid injection, shockwave therapy, ultrasonography, laser therapy, acupuncture, dry needling [10], topical glyceryl trinitrate patches [11], prolotherapy [12], Doppler ultrasound guided polidocanol injections [13], autologous blood and platelet-rich plasma (PRP) [14] may be utilised as adjuncts. For more severe tendon injuries (large tears or ruptures), surgical intervention is unavoidable; however, current surgical techniques do little to address the poor regeneration ability of tendon.

Recent advances in cell therapy concentrate on addressing the underlying pathology of tendon degeneration [15], emerging as a promising non-surgical management option for tendinopathies [16–21]. Among of them, autologous tendon cell injection (ATI) is a promising non-surgical treatment for tendinopathies. The procedure involves harvesting autologous tendon tissue, the isolation of the tendon cells, expansion under quality assured good manufacture practice (GMP) cell laboratory and the injection of the tendon cells via ultrasound guided into the degenerative tendon tissue [18–20].

This is the first review summarising and discussing the current progress and challenges in the clinical use of tendon-derived cell therapy. Concluding this, future perspectives for tendon-derived cell therapy and potential improvement by current basic knowledge are discussed.

## Tendon-derived cell therapy: the homologous use for tendon repair

Historically, well differentiated tenocytes were thought to be the only cell type present in tendons, but recent identification of tendon-derived stem cells (TDSCs) suggested that tendon also contains tendon progenitor cells that have similar stem cell characteristics [22, 23]. Thus, cells derived from tendon are generally deemed to contain both well differentiated mature tenocytes or/and less differentiated tendon-derived stem cells. In this review, we use the terms tendon cell or tendon-derived cell as the general term for differentiated tenocytes or/and TDSCs.

Homologous use of tendon-derived cell is considered to be an ideal process for treatment of tendinopathy. According to the Food and Drug Administration (FDA) Guidelines [24], homologous use such as tendon-derived cell therapy for tendinopathy can reasonably be expected to function appropriately, while non-homologous use such as bone marrow-derived stem cell (BMSC) and adipose-derived stem cell (ADSC) therapy (both of which are common treatments for tendon injuries [25–27]), has increased safety and efficacy concerns. This statement is supported by several studies. Youngstrom et al. provided a systematic comparison of TDSC, ADSC and BMSC using a bioreactor system, and showed that TDSCs exhibited a higher tenocytic gene expression profile and better mechanical strength among the three mesenchymal stem cells (MSCs) [28]. Comparisons between TDSC and BMSC isolated from rat have shown that TDSCs expressed higher stem cell marker Octamer-binding transcription factor 4 (OCT4), higher proliferation rate and clonogenicity than BMSCs [29]. Moreover, TDSCs also showed higher transcription levels of Tenomodulin, Scleraxis (SCX), collagen type I and decorin, suggesting that TDSCs are a better cell source for tendon regeneration. An in vivo study of patellar tendon defects showed that the TDSCs-repaired group had better biomechanical outcome with higher Young’s modulus than BMSCs-repaired group [30]. Similarly, using autologous TDSCs and BMSCs implantation for ruptured Achilles tendon repairs also confirmed that TDSCs could better restore mechanical properties of tendon with higher ultimate failure force and greater collagen synthesis [31].

Tendon-derived cell therapy has been used for treatment of late-stage tendinopathy and tear in different anatomical sites including elbow, gluteal and rotator cuff. The mechanisms of action are multi-factorial including (1) to replenish the local tendon cell population; (2) to promote tissue regeneration by counteracting the intrinsically slow healing process; and (3) to stimulate the production of growth factors [20] and the synthesis of matrix proteins (e.g., type I collagen) [16, 17, 32, 33]. Current clinical studies have not specifically identified their cell sources as TDSC or well-differentiated tenocytes and thus require further confirmation. Cell fate tracking in preclinical animal studies has shown that implanted autologous tendon-derived cells remain localised and integrated in the tendon tissue and promote tissue repair and regeneration via production of functional matrix proteins including type I collagen [17, 32]. Additionally, biodistribution analyses have ruled out systemic migration of tenocytic cells to organs such as liver, heart and lung post-implantation [32]. Proof-of-concept preclinical animal studies [16, 17, 32, 33] and clinical studies [18–21, 34, 35] demonstrated promising results regarding the healing of tendon structure, and significant clinical improvements in pain, strength and functional outcome measures (Table 1).

**Table 1:** Summary of clinical studies of tendon-derived cell therapy

Injuries	Duration of symptoms	Follow-up	Number of patients	Clinical outcome*	References
Lateral epicondylitis	29.24 months	4.5 years	16	*VAS* 5.73 to 1.21 [78%] *QuickDASH* 45.88 to 6.61 [84%] *UEFS* 31.73 to 9.20 [64%] *Grip strength score* 19.85 to 46.60 [208%] *MRI score* 4.31 to 2.87 *Patients satisfied with treatment* 93%	[19]
	31 months	1 year	20	*VAS* 5.94 to 0.76 *QuickDASH* 45.88 to 3.84 *Grip strength score* 20.17 to 37.38 *MRI score* 4.31 to 2.88	[18]
Gluteal tendinopathy	33 months	2 years	12	*VAS* 7.2 to 3.1 *OHS* 24 to 38.9 *SF-36* 28.1 to 43.3 *Patients satisfied with treatment* 8/12	[20]
Rotator cuff tendinopathy	12 months	1 year	1	*VAS* 1 *Oxford shoulder score* 47 *QuickDASH* 13 *MRI* partial-thickness rim-rent tear not detectable	[34]
	4 months	6 months	1	*MRI score* 5 to 1.33 *Internal rotation strength* 231-253 N Athlete returned to full training pain free and international level competition	[35]
	Chronic pain with five-month acute exacerbation	More than 12 months	1	Completely symptom-free Complete range of movement and returned to golf game without limitation	[21]

*In clinical outcome, A to B means pre-treated assessment A to post-treated assessment B, e.g., “VAS: 5.73 to 1.21” means pre-treated VAS is 5.73 and post-treated VAS is 1.21; [] means percentage of improvement

*VAS* Visual Analog Scale, *QuickDASH* Quick Disabilities of Arm, Shoulder and Hand, *UEFS* Upper Extremity Functional Scale, *MRI* magnetic resonance imaging, *OHS* Oxford hip score, *SF-36* short form 36

## Challenges in tendon-derived cell therapy

There are two major challenges in the development of tendon-derived cell therapy. Firstly, although clinical outcome on use of the ATI for treatment of midsubstance of tendinopathy and tear is promising, it remains unclear whether these cells are capable of replenishing tissues at different parts of tendons (MTJ, midsubstance of tendon, and enthesis). There is a need of developing precise tendon-derived cell therapy on injuries at different parts of tendons. Secondly, there are many factors contributing to the fitness of the tendon cells. Unfit tendon cells may undergo failure of in vitro expansion or be poorly functional for producing ECM. Here we have discussed these two major challenges.

### Challenge 1- Differentiation status of tendon-derived cells is a complex issue

Tendons consist of MTJ, midsubstance of tendon and enthesis [36]. The composition of ECM proteins contributes to the tensile property of tendons, which varies in different areas of the tendon. The main component of tendon is collagen. Several subtypes of collagen have been identified, including type I, type II and type III. Other components of ECM include 1% to 5% proteoglycans and glycoproteins, 2% elastin and 0.2% inorganic molecules [37].

**Fig. 1:**
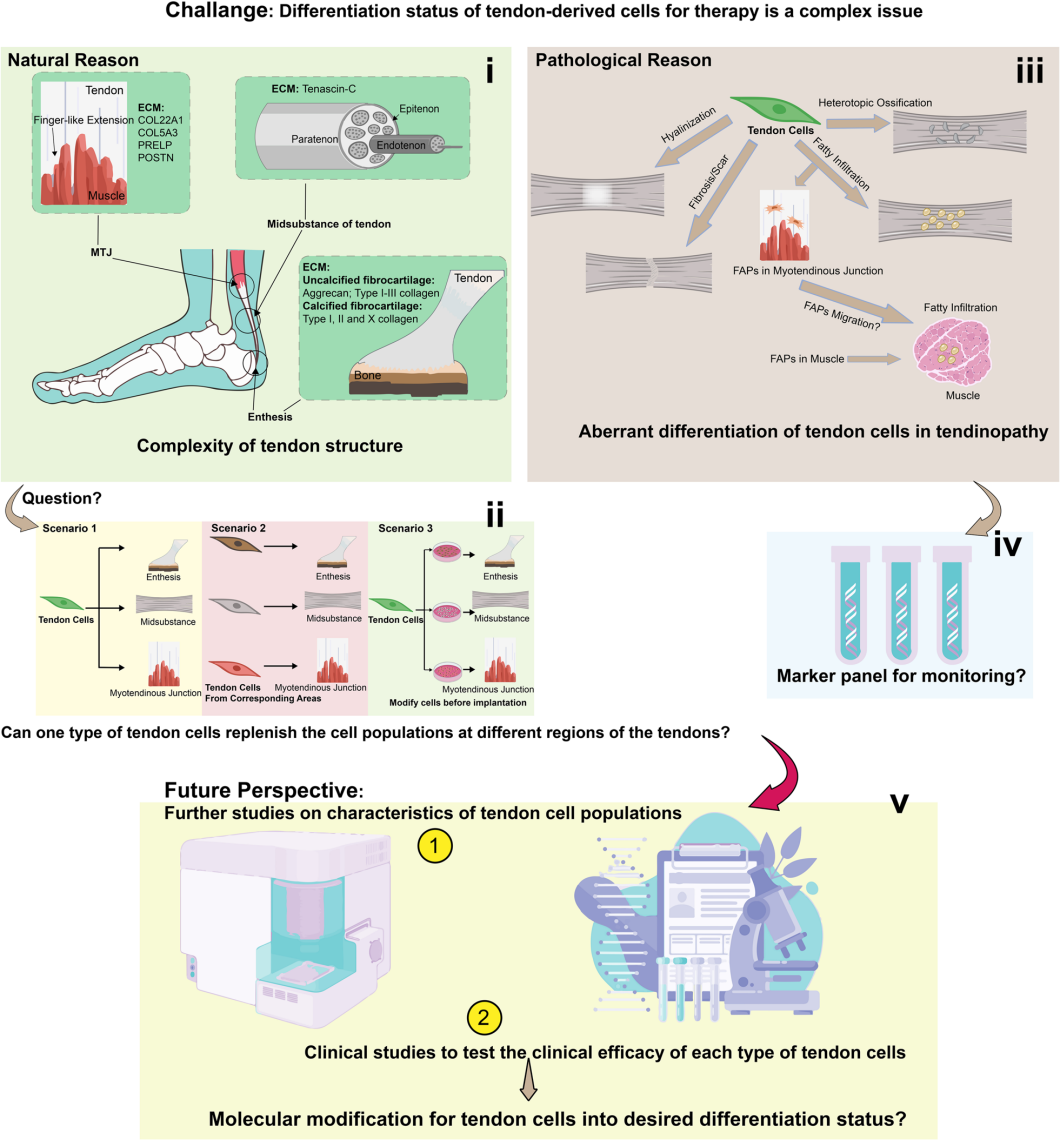
Challenge and future perspectives about differentiation status of tendon-derived cells for therapy. Natural reason of various differentiation status of tendon cells is the complexity of tendon structure **(i)**. A tendon consists of myotendinous junction (MTJ), midsubstance of tendon and enthesis. The different composition of extracellular matrix (ECM) proteins secreted by the tendon cells at different regions of tendon results in the niches difference for cellular differentiation, which leads to three possible scenarios for the tendon cell derived therapy at MTJ, midsubstance of tendon and enthesis (**ii**). (1) One type of tendon-derived cells can be used for tendon repair at MTJ, midsubstance of tendon and enthesis. (2) Specific types of tendon-derived cells are required for tendon repair at MTJ, midsubstance of tendon and enthesis. (3) One type of tendon-derived cells can be used for tendon repair at MTJ, midsubstance of tendon and enthesis after the modification by distinct cell culture protocols. Pathological reason of various differentiation status of tendon cells is the aberrant differentiation in tendinopathy (**iii**). Aberrant differentiation of tendon cells triggered by intrinsic and extrinsic signals can lead to heterotrophic ossification, fibrosis, hyalinisation and fatty infiltration (**iv**). Based on current findings on molecular signals, marker panel is an option for monitoring aberrant differentiation (**v**). Future perspective for this challenge needs further basic studies on characteristics of tendon cells and clinical studies on the clinical efficacy of different types of tendon cells. If certain differentiation status of tendon cells is essential, molecular modification for tendon cells into desired differentiation status is possible. FAP, fibro/adipogenic progenitor cell

ECM composition of MTJ, midsubstance of tendon and enthesis are unique (Fig. 1i). Proteomic analysis of the ECM components in MTJ and midsubstance of tendon revealed that type I collagen was expressed in both sites. MTJ was characterised by high expression of COL22A1, COL5A3, PRELP and POSTN [38], whereas midsubstance of tendon was characterised by high expression Tenascin C (TNC). Entheses expressed yet a different composition of ECM, in itself also different from uncalcified fibrocartilage, calcified fibrocartilage and bone. Uncalcified fibrocartilage was characterised by Aggrecan and type I–III collagen, while calcified fibrocartilage was characterised by type I, II and X collagen [39–42]. Different ECM production at different regions plays a critical role in tendon homeostasis with respect to different force adaptation and tissue surface attachment required at each location.

The distinct transcriptional mechanisms regulating the production of ECM in tendon cells at MTJ, midsubstance of tendon and enthesis are only partially understood. It remains unclear if tendon-derived cells from different parts of tendon are the same type. Thus, it raises the fundamental question of whether tendon-derived cells from a random site can be used for tendon repair at multiple sites (MTJ, midsubstance of tendon and enthesis); if specific types of tendon-derived cells are required for tendon repair at specific sites; or if one type of tendon-derived cell can undergo modification by cell culture method (e.g., bone morphogenetic protein 2—BMP2 for enthesis) before being using for cell therapy (Fig. 1ii).

In this section, we summarise and discuss the current understanding of the identity of tendon cells, cell differentiation at MTJ, midsubstance of tendon and enthesis and aberrant differentiation of tendon cells due to pathological conditions.

#### Blurred line between differentiated tenocyte and TDSC

Differentiated tenocytes and TDSCs are two major cell sources with different renewal capacity for tendon-derived cell therapy. However, as the isolation procedures and morphology of differentiated tenocytes and TDSCs are similar, identification of these two types of cells remains difficult [43]. As a result, in clinical studies of tendon-derived cell therapy, donor cells are the mixture of differentiated tenocytes and TDSCs. The difference in the efficacy of these two cell types for cell therapy remains unclear and requires further investigation, although several studies have provided insights on these two types of cells. Zhang et al. used micropipette to separate rabbit TDSCs and differentiated tenocytes and found that while nucleostemin, OCT-4 and stage-specific embryonic antigen-4 (SSEA-4) were highly expressed in TDSCs, it was lowly expressed in differentiated tenocytes [23]. Williamson et al. used differential adhesion to fibronectin substrates to isolate equine differentiated tenocytes and TDSCs and found higher expression of TSP-4 in differentiated tenocytes than TDSCs [44]. Lee et al. applied cloning cylinders to isolate murine TDSCs and differentiated tenocytes, showing higher expressions of Mohawk homeobox (MKX), SCX, CD73 and Nanog, and lower expressions of CD45, TSP-4, TNMD and TNC in TDSCs than differentiated tenocytes [22]. With the advances in gene sequencing technology, global transcriptome analyses on tendons have been employed to define the identities of differentiated tenocytes/TDSCs [45, 46]. Five subpopulations were found in both human healthy and diseased tendon samples. The five tendon subpopulations may represent different types of differentiated tenocytes and TDSCs at the midsubstance region of tendon. Tubulin polymerisation-promoting protein family member 3 (TPPP3) positive cells were found in one of the subpopulations which may represent one type of TDSCs [47]. Transcriptome analysis on mouse tendon suggests that Nestin^+^ cell population labels the TDSC population, whereas Nestin^–^ cell population labels the more differentiated tenocytes [46]. No data regarding the transcriptome profiles of tendon cells in MTJ and enthesis of human tendon are available yet.

#### Defined MSC markers may not fit for TDSC

TDSCs are considered the most ideal choice for cell therapy for tendon disease due to its self-renewal capabilities and homologous nature [43]. Purification of TDSCs requires detailed understanding of surface markers. TDSCs, first reported in human and mouse tendon tissues by two studies [48, 49] and later found in other animal models [23, 50, 51], are one type of MSCs located in tendon tissue and share some of the criteria with BMSCs and ADSCs established by the Mesenchymal and Tissue Stem Cell Committee of the International Society for Cellular Therapy [52] (Table 2). The minimal criteria for MSCs are: (1) adhere to plastic culture dish; (2) positive for CD73 (ecto-5’-nucleotidase), CD90 (Thy-1) and CD105 (endoglin), but negative for CD11b or CD14 (hematopoietic cells), CD19 or CD79a (B cells), CD34 (primitive hematopoietic progenitors and endothelial cells), CD45 (pan-leukocyte marker) and HLA-DR (MHC class II cell surface receptor) surface markers; and (3) be able to differentiate to osteoblasts, adipocytes and chondroblasts in vitro. TDSCs showed greater than 90% of CD73 and CD90 expression (43, 51) but with subtle differences in CD105 expression compared to MSC criteria (Table 2). Asai et al. demonstrated that there were two distinct cell populations, CD105 + ve (CD105 positive) and CD105-ve (CD105 negative), in mouse tendon [53]. The CD105-ve population showed superior chondrogenic potential, raising the possibility that CD105 + ve and CD105-ve may represent two different types of TDSC populations [53]. The difference in CD105 suggests that the minimal criteria for MSCs may not be suitable for application in TDSCs. Similar to BMSCs and ADSCs, TDSCs are consistently positive with CD44 (cell surface HA-binding glycoprotein) and negative for CD34, CD45 and HLA-DR, but exhibited differently in CD14 (Table 2). It remains unclear whether the difference in CD14 is due to contamination of hematopoietic cells or an intrinsic difference of TDSCs. Further data regarding the expression of CD11b or CD14 and CD19 or CD79a in TDSCs are required for the analysis. These studies indicated that TDSCs may have different cell surface identity compared to the current MSC criteria, in particular CD105 and CD14 (Table 2). For example, in injured tendon, TDSC contains larger CD105-ve populations [53]. Although standard indicators established for MSC still are used on TDSC by most researchers, further studies are required to investigate the subtypes and some subtle variations of TDSCs under distinct physio-pathological situations.

**Table 2:** Expression of CD markers in human BMSCs, ADSCs and TDSCs

MSC	Human BMSCs	Human ADSCs	Human TDSCs
CD73	100% [54]	96.12% [55]	100% [54]
	90.4% ± 5.1% [56]	92.4% ± 4% [56]	99.9% [57]
	98.7% [58]		99.6% [59]
	99.2% [60]		
CD90	100% [54]	95.54% [55]	100% [54]
	84.8% ± 4% [56]	99.53[61]	97.43% [48]
	96% [58]	99.67% [62]	94% [63]
	91.7% [60]	90.4% ± 3% [56]	99.11% [64]
			99.8% [57]
			99.8% [59]
CD105	100% [54]	95.08% [55]	100% [54]
	93.8 ± 4.6% [49]	98.76% [62]	0.1% [63]
	97.8% ± 0.6% [56]	93.8% ± 2.8% [56]	72.6 ± 22.9% [49]
	99.0% [58]		84.75% [64]
	99.6% [60]		92.6% [57]
			98.3% [59]
			Less than 90% [46]
CD44	100% [54]	100% [61]	99.82% [48]
	97.2% [58]	99.95% [65]	100% [54]
		80% [66]	93% [63]
		99.85% [62]	100% [64]
			99.9% [57]
CD34	0% [54]	0.26% [55]	0% [54]
	0.3 ± 0.4% [49]	1.05% [61]	0% [63]
	2.2% ± 0.3% [56]	0.01% [65]	0.9 ± 1.1% [49]
	0.455% [58]	3% [66]	0% [57]
	0.1% [60]	0.14% [62]	
		3% ± 1.1% [56]	
		0.01% [65]	
CD45	0% [54]	0.11% [55]	0% [54]
	2.7% ± 0.7% [56]	1.69% [61]	0% [63]
	1.07% [58]	0.03% [65]	0.15% [48]
	0.2% [60]	1.17% [62]	0.32% [64]
		3% ± 1.2% [56]	5% [57]
		0.01% [65]	
CD79a	–	–	–
CD19	1.08% [58]	0.67% [55]	–
	0.1% [60]	0.02% [65]	
		2.2% [67]	
CD14	0.499% [58]	0.06% [55]	12.12% [64]
	0.3% [60]	0.02% [65]	
CD11b	–	0.02% [65]	–
		1.3% [67]	
HLA-DR	1% [54]	0.19% [55]	0% [54]
	1.5% [58]	0.27% [62]	
	0.1 [60]	0.02% [65]	

*CD* Cluster of differentiation, *BMSC* Bone marrow-derived stem cell, *ADSC* Adipose-derived stem cell, *TDSC* Tendon-derived stem cell, *HLA-DR* Human leukocyte antigen-DR isotype

#### Limited knowledge on heterogeneity of tendon cells

Tendon cells can be extracted from different anatomical regions including enthesis, MTJ and midsubstance of tendon [48, 49, 63, 68]. Tendon cells from these different areas may express different sets of ECM proteins as they are unique with respect to their homeostasis requirements. Tendon cells in midsubstance of tendon express tendon-specific ECM proteins, while cells in tendon-bone or tendon–muscle junctions express additional specific proteins such as cell adhesion molecules to facilitate their attachment to bone and muscle [42]. Some studies have been done to characterise tendon cells, showing unique signatures of molecule expression in different regions of tendon. TDSCs extracted from human enthesis showed great chondrogenic potential [63]. Another study showed that TDSCs derived from MTJ showed lower expression of* Gdf 15 *(marker of tenogenesis) and *Vcam1 *(MSC-specific marker) than those derived from midsubstance of tendon in rat [68]. In mouse, SCX/SRY-box transcription factor 9 (SOX9)/GLI family zinc finger 1 (GLI1) marked the progenitor cells which contribute to the bone-tendon junction [69, 70]. As CD105-ve TDSCs may have more of a tendency to differentiate into cells at tendon-bone junction in mouse [53], further examination of GLI1, SCX and SOX9 expression in both CD105 + ve and CD105-ve cell populations may provide novel insights into subpopulations of TDSCs in forming different ECM. Unfortunately, there have been no studies on TDSCs isolated from MTJ in human. Utsunomiya H. et al. characterised the TDSCs derived from enthesis and midsubstance of tendon using cell surface markers [63] and found that human TDSCs derived from enthesis and midsubstance of tendon showed different expression of surface markers such as CD44, CD90, CD147 and CD166. There is a knowledge gap regarding comprehensive analyses of the surface makers of tendon cells derived from the enthesis, MTJ and midsubstance of human tendons. Further investigation of the identities of tendon cells could be better defined by comparison of transcriptome profiles and the surface markers of the cells isolated from MTJ, enthesis and midsubstance of tendons.

#### Distinct cell differentiation at midsubstance, enthesis and MTJ confers cell heterogeneity

Differentiation progress of tendon cells varies at enthesis, midsubstance and MTJ. During embryonic development, tendon cells can be derived from paraxial mesoderm (PM), lateral plate mesoderm (LPM) and cranial neural crest cells (CNCCs) [71, 72]. PM cells are condensed to form somites which are later segregated into different discrete domains—dermatome, myotome, syndetome and sclerotome. Tendon cells specifically originate from syndetome and are located between differentiating muscles and bones. LPM cells are condensed to form limb buds which further differentiate into muscles, cartilages, bones and tendons. The primordia-forming muscles, bones and tendons are spatially located next to each other in the trunk region. In contrast, the formation of tendons, muscles and bones are derived from different cell origins in cranial regions. Muscles and bones are mainly derived from cranial mesoderm, whereas tendons are specifically derived from cranial neural crest [72]. Neural crest is a transient multipotent cell population that arises from the neural plate border. The CNCCs migrate from cranial neural fold to form cartilages and tendons in the cranial regions and migrate along with the cephalic mesoderm which is finally invaded and circumscribed by CNCCs. The CNCCs and cephalic mesoderm share similar spatial arrangement to that in the trunk region. The CNCCs expressing SCX^+^ near the muscle cells become tendons. The tenoblast lineage differentiation in the trunk and cranial regions share similar differentiation programs with the initial establishment of SCX expression. In these processes, molecular signalling significantly contributes to program the cell differentiation at enthesis, midsubstance and MTJ.

**Fig. 2:**
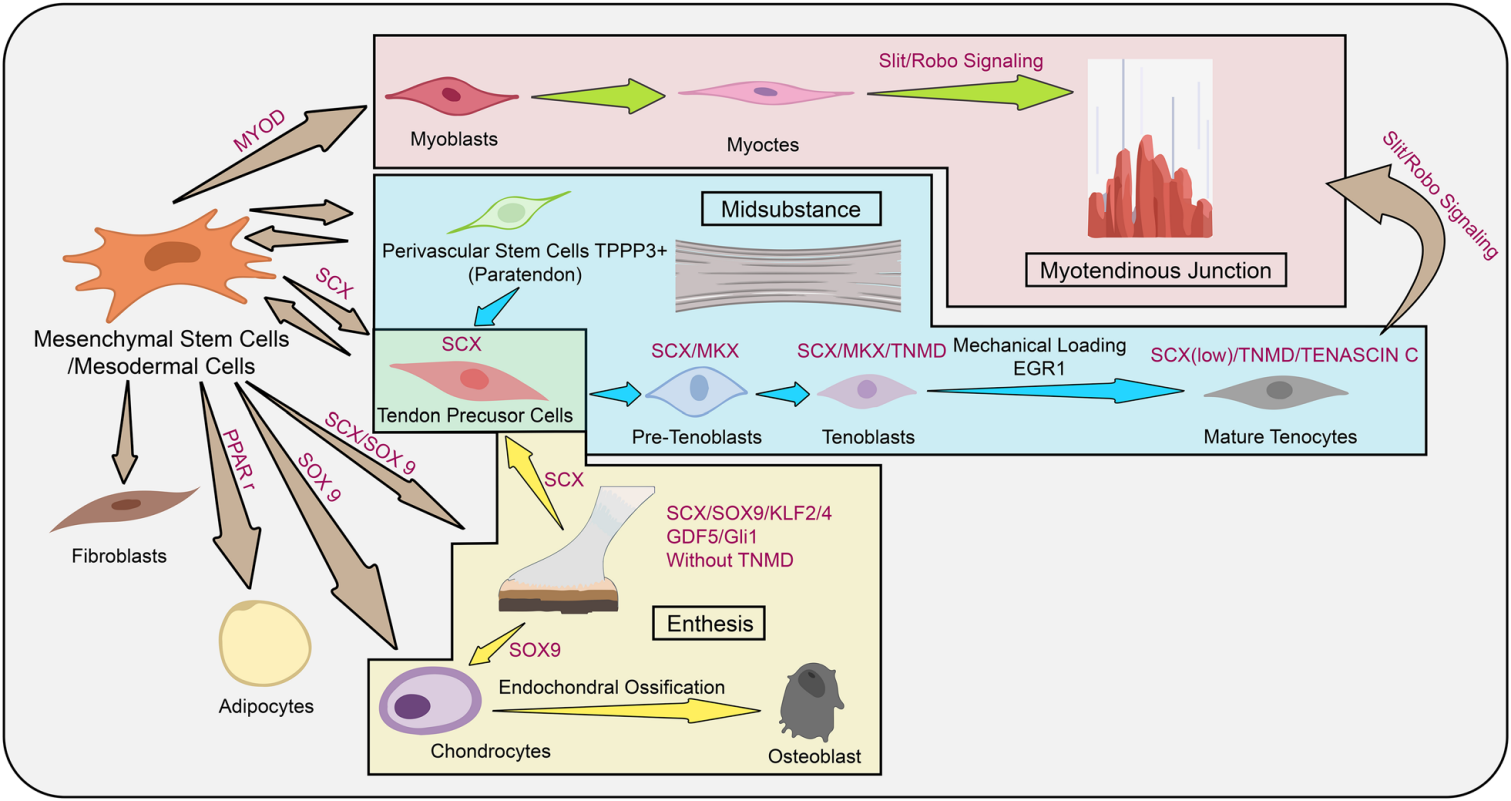
Cell differentiation at enthesis, myotendinous junction (MTJ) and midsubstance of tendon. Mesenchymal stem cells/mesodermal cells are capable of differentiating into fibroblasts, adipocytes, chondrocytes, osteoblasts and tenoblasts (premature tenocytes). RED panel shows the formation of MTJ by muscle and tendon interaction via SLIT/ROBO signalling. BLUE panel shows the differentiation of tendon precursor cells into mature tenocytes via multiple steps (pre-tenoblasts and tenoblasts) by upregulation of SCX, MKX, TNMD and/or TNC at different stages. YELLOW panel shows the SCX + /SOX9 + bi-fated progenitors at the putative enthesis regions and the progenitors are segregated into SCX + /SOX9 + cells to become tendon, chondrocytes and osteoblasts in the enthesis

The development of the midsubstance of the tendon is orchestrated by several crucial molecules. SCX, a basic helix–loop–helix transcription factor, marks the tendon cells or ligament cells and is the first marker expressed in tendon precursors in the tenoblast lineage [73]. In the early stage, tendon precursors, similar to the MSCs and TDSCs, are able to differentiate into chondrocytes, osteoblasts, adipocytes, myocytes and fibroblasts in the presence of extrinsic and intrinsic signals (Fig. 2). MKX, a member of the three amino acid loop extension superclass of atypical homeobox, is expressed with the SCX at similar time point [74]. We propose that SCX^+^/MKX^+^/SOX9^−^ cells (SOX9 will be discussed in next paragraph) are lineage-restricted pre-tenoblasts that are committed to tendon cell lineage. Later, both SCX and MKX activate Tenomodulin (TNMD) expression to form more mature tenoblasts [75–77]. With contributing mechanical stimulation by muscle contraction and relaxation, EGR1 and TNC are upregulated in fully differentiated tenocytes [78, 79], whereas SCX expression is downregulated in the mature tenocytes [43]. Relatively high level of Tenascin C in midsubstance of tendon helps differentiation from enthesis and MTJ. TPPP3^+^ perivascular stem cells in paratenon derived from mesodermal cells/MSCs can express SCX to become tendon precursors that migrate to the injured tendon and become mature tenocytes through the similar pathway (Fig. 2) [47].

In enthesis, where fibrocartilage and tendon tissue co-exist, there is a more complex regulation network. Apart from SCX, SOX9, a SRY-like HMG box-containing transcription factor, marks chondrocytes and is the major regulator for chondrogenesis and also plays an important role in enthesis establishment [73, 80, 81]. Interestingly, tendon cells can be converted to chondrocytes by forced expression of SOX9 in vitro [82]. In the early stage of musculoskeletal development, SCX and SOX9 are co-expressed in progenitor cells (Fig. 2). A lineage tracing experiment found that there were bi-fated progenitors SCX^+^/SOX9^+^, which could contribute to the interface between cartilage and tendon at enthesis [83]. The bi-fated progenitors eventually differentiate into chondrocytes and tenocytes through the segregation of lineage into SCX^+^/SOX9^−^ and SCX^−^/SOX9^+^ cells to form different zones at the attachment site from 11.5dpc to 13.5dpc in mouse [83, 84]. In later stages (14.5dpc in mouse), bi-fated attachment cells (mainly GLI1^+^) regulated by KLF2/4 and GLI1 are still present in enthesis and are important for enthesis regeneration [70, 84, 85]. Tendon precursors expressing SCX follow the differentiation pattern similar to that at the midsubstance. SOX9^+^ progenitors differentiate into chondrocytes that express Col2a1 and other chondrocyte-specific ECM proteins which constitutes the specific zones at enthesis [80, 86]. Proliferating chondrocytes further develop into hypertrophic chondrocytes that express COLX. Hypertrophic chondrocytes are able to transdifferentiate into osteoblasts expressing type 1 collagen [87]. These proteins contribute to the unique ECM composition at the different zones of enthesis.

MTJ can be found in invertebrates, such as drosophila, suggesting a conserved pathway for the formation of MTJ [88]. MTJ formation has two stages, including muscle–tendon establishment and muscle–tendon adhesion. The establishment of MTJ is not due to the formation of the bi-fated progenitors like those observed at enthesis. In contrast, the establishment of MTJ is the result of the tissue interaction between myogenic progenitors and tendon progenitors at the embryonic stages (Fig. 2). The migrating embryonic muscle progenitors expressing Slit receptor Roundabout (ROBO) are attracted by SLIT secreted by tendon progenitor cells to colonise at the MTJ [88, 89]. Tendon progenitors also secrete the muscle arrest protein Leucine-rich tendon-specific protein (LRT) to stop the filopodia formation of muscle [90]. During the stage of muscle–tendon adhesion, muscle cells and tendon cells can express integrins, ILK, TALIN, MSK and focal adhesion kinase (FAK) and paxillin, which acts as adhesion clues at MTJ [91]. However, the transcriptional program for the activation of COL22A1, COL5A3, PRELP and POSTN at MTJ remains to be elucidated.

#### Current protocol of tenogenesis does not consider heterogeneity

In vitro expansion of tendon cells is an essential step in tendon-derived cell therapy. With regards to cell therapy efficacy, maintenance of tenocytic phenotype and promoting the tenogenic differentiation of tendon cells are pivotal. Especially for TDSCs, which possess the trilineage differentiation potential as previously described, its differentiation status is close to the tendon precursor in the tenoblast lineage (Fig. 2). Regulation of tenogenic differentiation to TDSCs is a complex process involving numerous chemical and biomechanical factors. TDSCs have been known to be able to undergo spontaneous tenogenic differentiation but be inhibited under TGF-β1 induction [92, 93]. Modulation of activity of TGF-β family can regulate tenogenic differentiation of TDSCs [94, 95]. In addition, other molecules like growth/differentiation factors (GDFs), connective tissue growth factor (CTGF), biglycan, interleukin-10, cystic fibrosis transmembrane conductance regulator (CFTR) and SPARC can also regulate the process of tenogenesis of TDSCs [96–99].

Tenogenic differentiation of TDSCs involves a biomechanical network contribution, which can be employed in tendon-derived cell therapy. The components in the biomechanical network include loading direction (uniaxial or biaxial), loading interval (cyclic or constant), culture methods (2D or 3D) and stiffness of culture which affect the tenogenic differentiation of TDSCs. Despite the inconsistency of loading regimes used in different studies, most researchers reached the consensus that mechanically loaded TDSCs are more motivated into tenogenic differentiation and showed tenocytic phenotype as compared to static TDSCs [100–103]. Bi et al. showed that transplanted TDSCs with scaffolds could form tendon-like tissue evidenced by specific parallel collagen fibres and enriched type I collagen [48].

Tenogenesis can be evaluated by the expression of SCX, MKX, early growth response 1(EGR1), TNMD, collagen type I, Tenascin C, decorin, biglycan and fibromodulin [48, 74, 82, 103–105]. Evaluation of tenogenesis of tendon cells prior to injection to patients is a necessary contributor to treatment efficacy.

#### Aberrant differentiation of tendon cells in tendinopathy

Due to the changes in microenvironment and abnormal mechanical loading seen in tendinopathy, aberrant differentiation occurs in tendon cells, mainly TDSCs, which can in turn causes pathological changes in tendon matrix. These include fibrosis, heterotopic ossification (HO), fatty infiltration and hyalinisation. All these changes in matrix are associated with the changes to TDSC cell fate, which may affect therapeutic effects if isolating and differentiating them improperly.

Fibrosis -Tenocytes in injured tendons can undergo programmed cell death after loading deprivation, and damage-associated molecular patterns (DAMPs) proteins are upregulated within hours of this damage [106–109]. Cell death leads to the activation of stromal cells and immune cells that brings the tendon tissue into an inflammatory state. Here, monocytes are turned into macrophages to remove apoptotic tenocytes and ECM debris. Days to weeks later, tendons proceed to a stage whereby TDSCs start to express SCX. The SCX^+^ TDSCs migrate from the sheath into the lesion, differentiate into tenocytes to replace dead tenocytes and release collagen III and fibronectin to temporarily repair ECM [110–114]. During this repair process, a portion of the SCX^+^ tendon cells will express alpha smooth muscle actin (αSMA), a myofibroblast marker in response to inflammatory signals [115]. Myofibroblasts are specialised cell types responsible for fibrosis during wound healing.

S100a4 (also known as Fsp1*, *Mts1*, *Pk9a) is a member of the S100 family of EF-hand Ca^2+^-binding proteins and a key regulator for tissue fibrosis [115]. S100a4 is expressed in both uninjured and injured tendons, but the S100a4^+^ cell population expands during tendon healing. Depletion of S100a4 has been proven to reduce fibrosis during tissue wound healing in other organs [116, 117]. Depletion of S100a4 in tendon improves the morphology and mechanical property of the tendons by reducing the myofibroblast population. Lineage tracing suggests that about 65% of αSMA^+^ cells at the injury site originated from S100a4^+^ cells [115]. This evidence supports the suggestion that S100a4 acts as the upstream mediator of αSMA, even though S100a4 expression is lost during the transition from TDSCs to SCX^+^αSMA^+^ cells, with this increased expression of SCX^+^S100a4^+^ cells leading to fibrotic tendinopathies (Fig. 1iii).

Heterotopic ossification (HO) -HO is a pathological condition in which ectopic bones are formed in tissues such as tendons. HO can occur in trauma (tHO) or hyperactive BMP conditions (bHO) (Fig. 1iii). Lineage tracing has confirmed that SCX descendant cells contribute to both tHO and bHO [118]. Markers for endochondral ossification, Osterix (OSX) and SOX9, were co-expressed in SCX descendant cells during HO formation in tendons, suggesting that endochondral ossification rather than intramembranous ossification contributes to HO. TDSCs have been demonstrated to possess osteogenic and chondrogenic differentiation capacity [48] and tendon cells can be converted into chondrocytes by overexpression of SOX9 [82]. Therefore, HO formation in tendons can be caused by aberrant differentiation of TDSCs through upregulation of SOX9. SCX downregulation in TDSCs can also induce chondrogenesis and osteogenesis which may finally lead to HO [110, 119]. However, it remains unclear whether SCX is downregulated during HO formation [118].

Fatty infiltration -Fatty infiltration is frequently observed in muscles after tendon tears such as rotator cuff tendon tears. The degree of fatty infiltration can be assessed by computerised tomography (CT) scan, magnetic resonance imaging (MRI) and ultrasound [120]. The origin of fatty infiltration is largely thought to be through adipocyte differentiation of the PDGFRα( +) fibro/adipogenic progenitor cells (FAPs) residing in muscle [121]. However, fatty infiltration seems to progress from MTJ into muscle after tendon injury, raising the possibility that the signals or cells from tendon may contribute to fatty infiltration in MTJ and/or muscles [122] (Fig. 1iii). A search of literatures unexpectedly identified a study showing similar PDGFRα^+^ FAPs present in tendons. The gene expression analysis of the TPPP3^+^/PDGFRα^+^ stem cells isolated from tendons surprisingly revealed two possible fates—tendon fibro/adipogenic progenitor (T-FAPs) cells and tendon cells. PDGFRα is particularly enriched in the T-FAPs, which is similar to the cell lineage FAP in the muscle [47]. Furthermore, fatty infiltration in tendon was detected as well [123]. Further research into whether T-FAPs can contribute to the fatty infiltration in injured tendons will provide interesting insights into the pathogenesis of tendon tears (Fig. 1iii).

Hyalinisation -Tissue hyalinisation refers to the conversion of stromal connective tissue into a homogeneous and glassy tissue which is mainly composed of acidic protein synthesis and few nuclei. Characteristically, it appears to be pink in colour after being stained with eosin. The glassy eosinophilic proteins are thought to be hyaline or hyaline-like materials such as type IV collagen, laminin, proteoglycans and hyaluronic acid. Hyalinisation acts as a hallmark for tissue degeneration. For example, hyaline arteriolosclerosis is associated with ageing [124] and is also one of the criteria to assess tendon ageing and degeneration [125]. Apoptosis is a key factor for hyalinisation in tendons [126] (Fig. 1iii), however, a recent study suggested that ageing tendon cells are more prone to differentiate into hyaline secreting fibrochondrocytes and mineralised fibrochondrocytes due to lower oxygen level as a result of decreased tendon vascularisation [127].

Together, we propose a panel of markers on aberrant tendon cell differentiation including S100a4/αSMA for fibrosis, OSX and SOX9 for HO; TPPP3/PDGFRα for fatty infiltration; type IV collagen, laminin, proteoglycans and hyaluronic acid for hyalinisation. These may enable to use as negative markers to monitor the differentiation status of the tendon-derived cells in vitro in tendon-derived cell therapy (Fig. 1iv).

### Challenge 2- There are factors affecting the fitness of tendon-derived cells for therapy

**Fig. 3:**
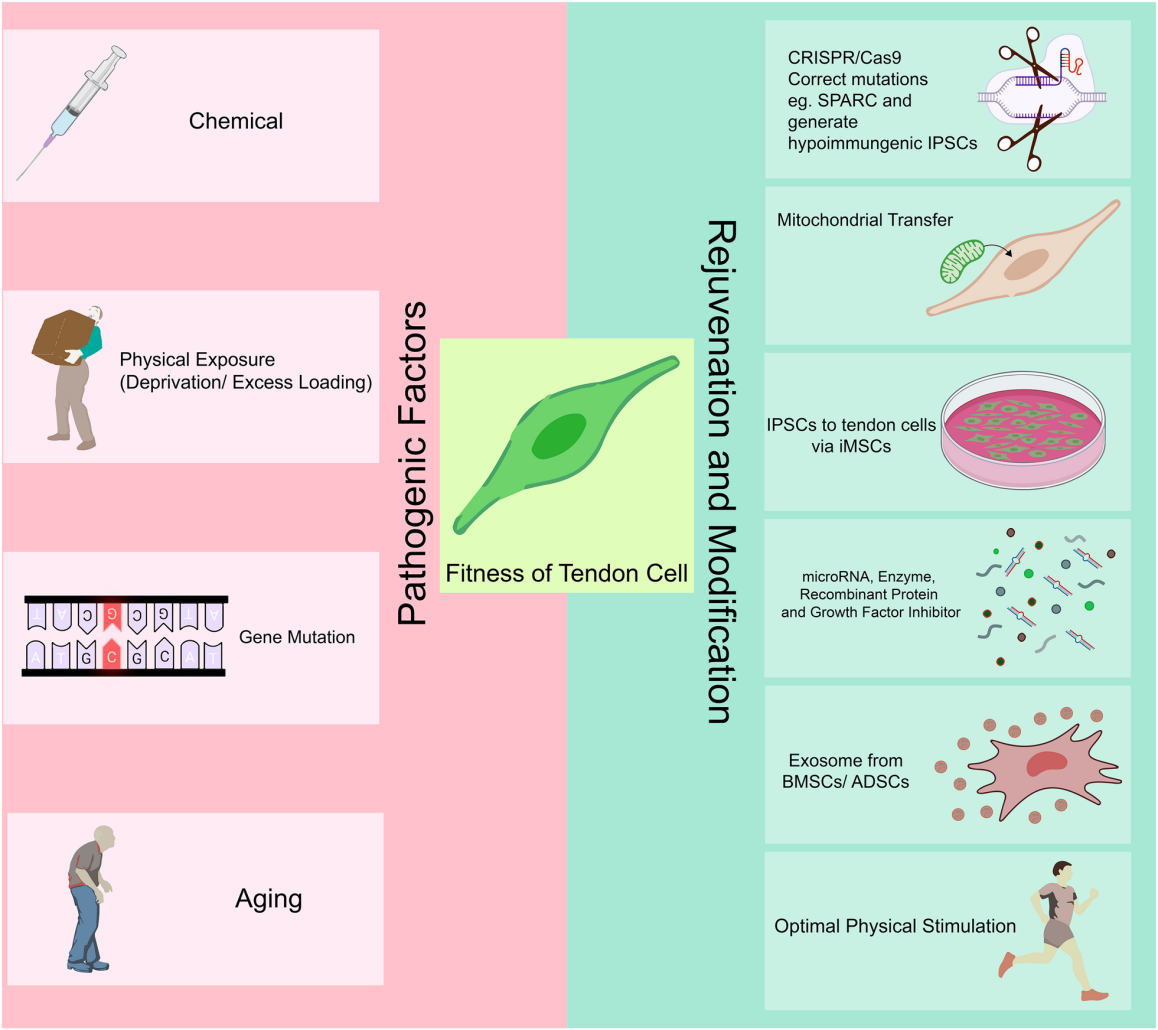
Pathogenic factors affecting fitness of tendon cells and potential strategies for rejuvenation and modification of tendon cells. Left panel summarises the pathogenic factors: chemical (e.g. Botox/Antibiotics), physical exposure (deprivation/pathological loading), gene mutations and ageing. Right panel summarises possible strategies to rejuvenate and modify TDSCs via (1) CRISPR/Cas9 (2) mitochondrial transfer (3) IPSCs to TDSCs via iMSCs (4) microRNA/enzyme/recombinant proteins/growth factor inhibitors (5) paracrine factors from BMSC/ADSC (exosomes) (6) optimal physical stimulation. BMSC, bone marrow-derived stem cell. ADSC, adipose-derived stem cell. TDSC, tendon-derived stem cell. IPSC, induced pluripotent stem cell. iMSC, IPSC-derived mesenchymal stem cell

As cell sources for tendon-derived cell therapy are commonly autologous, the overall health of the patient also affects the therapy efficacy. Tendon-derived cells become less efficacious in patients who are aged 65 years and over, genetic predisposition to tendinopathy, pathologically mechanically loaded tendons or exposure to certain chemicals (Fig. 3).

#### Ageing

Tissue ageing is the primary culprit leading to cell senescence [128]. The functional capacity of tendon cells declines with increasing cell passage number in vitro. Understanding of the mechanism regulating the tendon tissue ageing and senescence of tendon cells can provide a better insight into tendon diseases and their related treatments. Many studies have demonstrated the morphological changes of TDSCs isolated from aged tendon tissue. Compared to TDSCs isolated from young tendon tissue, TDSCs from aged tissue are larger and rounder and also display increased cell stiffness which might be due to the abnormal actin filaments accumulation and slower turn-over of ECM components [59, 129]. In addition, cell proliferation, colony formation capacity and differentiation capacity are compromised in aged TDSCs [130–132]. The phenotypes of TDSCs isolated from aged tendon tissue are mostly due to cellular senescence. Senescent cells are characterised by shortened telomeres, overexpression of senescence-associated β-galactosidase (SA-β-gal), increased expression of p16^INK4a^, p53 and p21^WAF1/Cip1^ [133]. Increased expression of p16^INK4a^ and p21^WAF1/Cip1^ were found in TDSCs from aged and degenerative Achilles tendon [93, 130]. A recent study showed that TDSCs from stress-deprivation induced atrophic tendon exhibited cellular senescence with intranuclear p53 accumulation [102]. Cellular senescence of TDSCs totally abolished the differentiation towards different lineages [102].

#### Risk predisposition

Certain genetic factors can increase an individual’s risk for tendon disorders. Tendon cells possessing germline mutations or their derivatives (e.g., differentiated tenocytes) may not be suitable as donor cells for cell therapy. Examples include mutations or derivatives of COL5A1, MIR608 [134], TIMP2, MMP3 [135], TNC [136], DEFB1, FGFR1, FGF10 [137], CASP8 [138], GDF5 [139], FGF3, BMP4 [140], ESRRB [141], FCRL3 [142], SASH1, SAP30BP [143], rs71404070 located next to cadherin8 [144], COL11A1 [145], ADAMTS14 [146], ACAN, BGN, DCN, LUM-DCN [111], COL1A1 [147], COL12A1 [148], MMP12 [149], COL3A1 [150], VEGFA [151], FAM111B [152], COL5A3 [153], FBN2 [154] and SPARC [155]. Although the majority of these studies only revealed an association between genetic factors and tendon diseases, with some of them representing contradictory conclusions [150, 156], our recent study on identification of a mutation of SPARC in patients with anterior cruciate ligament (ACL) and rotator cuff (RC) injuries [155] has suggested that use of autologous TDSC may not be suitable for treatment of these cohort of patients. Sequencing of the human *SPARC* gene in patients with a history of tendon injuries revealed that a missense mutation 388 T > G in *SPARC* gene with an allelic frequency of 5.52% (odd ratio 8.93) was associated with RC and ACL injuries, suggesting that this mutation is associated with tendon injuries.

#### Physical stimulation

Physical stimulation has been considered as a main contributing factor in tendon homeostasis [157, 158]. Thus, its effects on donor tendon cells may affect therapy efficiency. High load with repetitive mechanical stimulation is seen in most tendinopathies [159, 160], with obesity being shown to be detrimental on tendons [161]. Deprivation of mechanical stimulation to tendons has attracted increased attention in research recently. One study of TDSCs isolated from loading-deprived group by Botox injection displayed significant reduction in proliferation, clonogenicity and differentiation capacity [102]. Botox is a well-known neurotoxin that has been used for the treatment of a variety of musculoskeletal disorders (e.g., cerebral palsy, abnormal *tibialis posterior* and *gastrocnemius-soleus* contraction) [162, 163]. It acts by blocking the release of acetylcholine from cholinergic nerves into neuromuscular junction, leading to the reduction of muscle contraction [164]. Recently, it has been reported that Botox can be used for treatment of tendinopathy or tendon injuries [164]. The reduction of muscle activity induced by Botox has been demonstrated to be effective in relieving symptoms in tendinopathy and prevent tendon from muscle-contraction raised re-injury [165]. However, regardless of its specific use in muscle or tendon-related disorders, intramuscular injection of Botox results in either full or partial muscle relaxation and subsequent mechanical deprivation on related tendons. Insufficient mechanical loading has been shown to adversely affect tendon homeostasis and fitness of tendon cells [166]. One study using intramuscular Botox injection into *vastus lateralis* muscle to create a mechanically deprived patellar tendon model showed the adverse effects of tendon atrophy and collagen degeneration after Botox injection [102]. The senescent phenotype of TDSCs was found to be mediated by PTEN/AKT pathway of TDSCs from Botox injection group. The Botox chemical injection could indirectly affect the plasticity of TDSCs [102].

#### Chemical exposure

A number of chemical exposures are related to tendon disorders and thus potentially impact tendon cells function [167]. Fluoroquinolone antibiotics (including the commonly used ciprofloxacin, gemifloxacin, levofloxacin, moxifloxacin, norfloxacin and ofloxacin) have recently gained increased attention in tendinopathy [168, 169]. Fluoroquinolones inhibit the gyrase and topoisomerase of bacteria [170]. Unfortunately, similar enzymes exist in mitochondria. Fluoroquinolone has been confirmed to inhibit Topoisomerase II in mitochondria [171], which is required for initiation of mitochondria DNA (mtDNA) replication. Therefore, they have the ability to induce mtDNA depletion, mitochondrial dysfunction and oxidative stress in mammalian cells [172]. The US Food and Drug Administration has issued a Boxed Warning for fluoroquinolone in July 2008 due to the increased risk of tendinitis and tendon rupture [173]. Further to this, fluoroquinolones may induce oxidative stress and mitochondrial dysfunction in donor tendon cells and therefore impair tendon-derived cell therapy efficiency.

## Future perspectives of tendon-derived cell therapy

### Future perspectives for challenge 1

The characteristics of cell sources utilised in tendon-derived cell therapy remains unknown. Here, we discuss the further strategies in terms of tenogenic differentiation status and spatial heterogeneity of tendon cells to tendon-derived cell therapy.

#### Defined differentiation status of tendon cells for precise tendon-derivedcell therapy

The difference of proliferation rate of tenocytes and TDSCs is controversial and remains unclear [23, 43]. After the expansion of tendon-derived cells, we postulate that there are comparable amounts of tenocytes and TDSCs. Therefore, the tenogenic differentiation status of TDSCs should be considered in terms of therapeutic effects. Further determination of differentiation status of tendon cells can provide the clues to the efficiency and applicability of this cell therapy.

Cell hierarchy analyses at different culturing times using single-cell transcriptome should allow us to identify the tenogenic markers for tenocytes and TDSCs and the shift of cell populations prior to tendon cell implantation (Fig. 2). By detecting the expression levels of SCX/MKX, ACX/MKX/TNMD and SCX/TNMD/TNC, we will be able to evaluate the differentiation status of tendon-derived cells and the portions of tenocytes and TDSC in the cell mixture. Subsequently, the differentiation status and the clinical outcomes can be correlated. Based on this, tendon cells can be engineered to specific differentiation status by biochemical or biomechanical means prior to implantation to maximise the therapeutic effect [103, 174, 175]. These clinical and biological studies will be favourable in providing an insight into the treatment efficacy orchestrated by the differentiation status of tendon cells and also assist in developing a guideline for tendon-derived cell therapy with potential for individual patient customisation (Fig. 1v).

#### Clinical significance of  heterogeneity in tendon cells

Tendon cells exhibit different functionality in different areas, which is contributed to by specific ECM characteristics [77, 83, 176] (Fig. 1). Therefore, it is great of importance to have an insight as to whether tendon cells from different regions will have the same therapeutic effect and if so, under what mechanism tendon cells from different regions contribute to pathogenesis of tendinopathy.

Firstly, more in vivo studies should be conducted to evaluate the therapeutic efficiency of differently located tendon cells. In the aforementioned sections, tendon cells from distinct regions have been known to be involved in different molecular pathways (SCX/TNMD/TNC, SCX/SOX9 and SLIT/ROBO signalling pathway) (Fig. 2). Spatially heterogenetic tendon cells should be sorted and implanted in tendinopathy models. Post-implantation evaluation should be conducted to evaluate: (1) the restoration of normal tendon tissue on histological level (cell density, inflammation, collagen deposition, etc.); (2) functional recovery (range of limb motion, pain score, etc.) and (3) imaging correlation of tendon healing. This correlation of differently located tendon cells and therapeutic outcomes will clarify the difference or similarity of the therapeutic effects that the spatial heterogeneity of tendon cells might have impact on (Fig. 1v).

Secondly, even if we clarify the understanding of therapeutic outcomes of differently located tendon cells, the underlying mechanism is also worth elucidating. To gain these insights, cell tracing can be employed to identify the cell fate and key molecular alteration during the post-implanted healing process. Alternatively, single-cell transcriptome is another robust method in the of study tendon cells pre- and post-implantation. Full understanding of spatial heterogeneity will lead to a large improvement in tendon-derived cell therapy as well as to establish a valuable knowledge about the physiology of tendon healing.

The role of tendon cells in tendon-derived cell therapy is yet to be explained clearly. By addressing those questions, it is possible to construct a clinical guideline for tendon-derived cell therapy. If donor cells are more systematically evaluated before implantation, patients can receive standardised therapeutic benefits of maximal efficacy.

### Future perspectives for challenge 2

Despite the great achievements of tendon-derived cell therapy, ageing, genetic risk predisposition, pathological physical stimulation and chemical exposure can pose significant hurdles on its future development. To overcome this, we summarise and discuss the possible strategies to address the fitness of tendon-derived cells for therapy (Fig. 3).

#### *Rejuvenating tendon cells* via *mitochondrial implantation*

Mitochondria are the main powerhouse to supply energy for every cellular activity. Mitochondrial dysfunction is found in a range of diseases and is associated with cellular ageing processes. Naturally occurring and active mitochondrial transfers have been observed in vitro and in vivo as a way of tissue repair mechanism, such as in brain injury, stressed osteocytes, pulmonary injury and heart injury [177–179]. The mitochondrial transfer rejuvenates the target cells by relieving their oxidative stress, hypoxia stress and other cellular stresses [179]. Currently, a clinical trial is underway for the treatment of Pearson syndrome, a bone marrow failure disorder, has been initiated by enriching autologous CD34 + cells with blood-derived mitochondria (NCT03384420, clinicaltrials.gov). A recent study has connected the health of mitochondria and hypoxia stress to the rotator cuff injury [180]. Given the promising data in other tissues, mitochondrial transplantation for repairing tendon injury by local injection or centrifugation has been recently tested [181]. It showed that mitochondrial transplantation lessened dysregulation of oxidative stress and mitochondrial membrane potential and therefore could serve as a promising way to improve tendon-derived cell therapy.

#### Attenuation of cell senescence by agents

Cell senescence is a stable cell cycle arrest associated with ageing [182]. Several recent studies have attempted to attenuate the senescence of tendon cells in aged or injured group initially through the understanding of the mechanism of senescence of tendon cells. They proposed to attenuate senescence by microRNA, enzymes, recombinant proteins, growth factor inhibitor and physical stimulation. Compared to young TDSCs, CITED2 nuclear protein expression was lower in aged TDSCs and downregulation of CITED2 further potentiated the TGF beta 2-mediated senescence [130]. This study suggested that overexpression of CITED 2 or SB525334, an inhibitor of TGFβ receptor kinase, can prevent cell senescence. Similar to CITED 2, CTGF was found to be downregulated in aged TDSCs [183]. CTGF could induce the expression of BMP12, which is one of the key factors for tenogenesis. By applying recombinant protein CTGF in the aged TDSCs, less senescent TDSCs were observed, supporting that recombinant CTGF can rejuvenate the aged TDSCs. The other studies showed that the enzyme PIN1 (peptidyl-prolyl cis–trans isomerase NIMA-interacting 1) and the transcription factor forkhead box P1 (FOXP1) can prevent TDSCs from senescence in vitro [130, 184, 185]. In addition, recent studies further demonstrated that by overexpression microRNA miR-135a or inhibition of miR-124 could efficiently prevent TDSCs from senescence and maintain tenogenic differentiation [186, 187]. Furthermore, optimal physical stimulation can prevent TDSCs from undergoing cell senescence and degeneration [102]. Cyclic physical stimulation can prevent the degeneration of Achilles tendon [188]. All these studies contribute to the toolbox for rejuvenating aged TDSCs by attenuating cell senescence.

#### Exosomes to enhance treatment efficacy

The paracrine effects between tendon cells and BMSCs were first explored by indirect co-culture of BMSCs and tendon cells at 1:1 in transwell [189]. It was shown that tendon cells could effectively promote the proliferation of BMSCs after 3 days co-culture. All tenogenic-related genes (type I collagen, type III collagen, SCX and Tenascin C) were upregulated in BMSCs and TDSCs co-culture compared to BMSCs only group [189]. Interestingly, a recent study showed that TDSCs can uptake BMSCs-derived exosome and exhibited greater proliferation capacity, migration and tenogenic differentiation in vitro [190]. Further animal experiments showed that BMSC-derived exosome embedded in fibrin can be picked up by TDSCs for rat patellar tendon defect. Postoperative evaluation showed BMSC-derived exosome-treated group has enhanced expression of Mohawk, Tenomodulin and type I collagen as well as TDSCs migration [190]. Furthermore, the mechanical property of BMSC-derived exosome-treated group was superior to that of the control group [190]. This study provides invaluable insights on the exosome-mediated paracrine effect between TDSCs and BMSCs.

ADSC derived exosomes were identified in in vitro culture system to serve as a signalling messenger in paracrine signalling between TDSCs and ADSCs. Enhanced proliferation and differentiation in TDSCs were observed after TDSCs uptook exosomes from ADSCs [191]. Enhanced differentiation was evidenced by increased calcium formation under osteogenic induction and lipid droplet formation under adipogenic induction compared to TDSCs without ADSC-exosome [191]. To validate the effectiveness of ADSC-exosome on tendon repair, ADSC-exosome contained hydrogel was implanted into rat rotator cuff defect model. Histological examination revealed the improved collagen orientation and enthesis at 4 and 8 weeks in ADSC-exosome injection group compared to PBS injection [191]. The overall mRNA levels from repaired rotator cuff showed that tenogenic (*Tenascin C*, *Tnmd* and *Scx*), osteogenic (*Runx2*) and chondrogenic markers (*Sox9*) were also increased in ADSC-exosome injection group compared with PBS injection group [191]. The subsequent biomechanical test consistently showed higher maximal tensile force in ADSC-exosome group [191]. Another group performed ADSC-exosome injection to repair chronic rotator cuff tears in a rabbit model [192]. After 18 weeks injection, ADSC-exosome injection group showed lower fatty infiltration, which can adversely affect the healing results, and comparable mechanical strength as the saline-injected control group. Better fibrocartilage formation and more mature vessel infiltration were shown in enthesis. ADSC-exosome injection group also showed higher ultimate failure load, stiffness and stress than saline control group [192]. These studies suggest that treating tendon-derived cells with exosomes extracted from BMSCs or ADSCs could be a potential way to enhance proliferation and differentiation of tendon-derived cells.

#### iMSCs derived from induced pluripotent stem cells (IPSCs) as a way to produce rejuvenated tendon cells

**Fig. 4:**
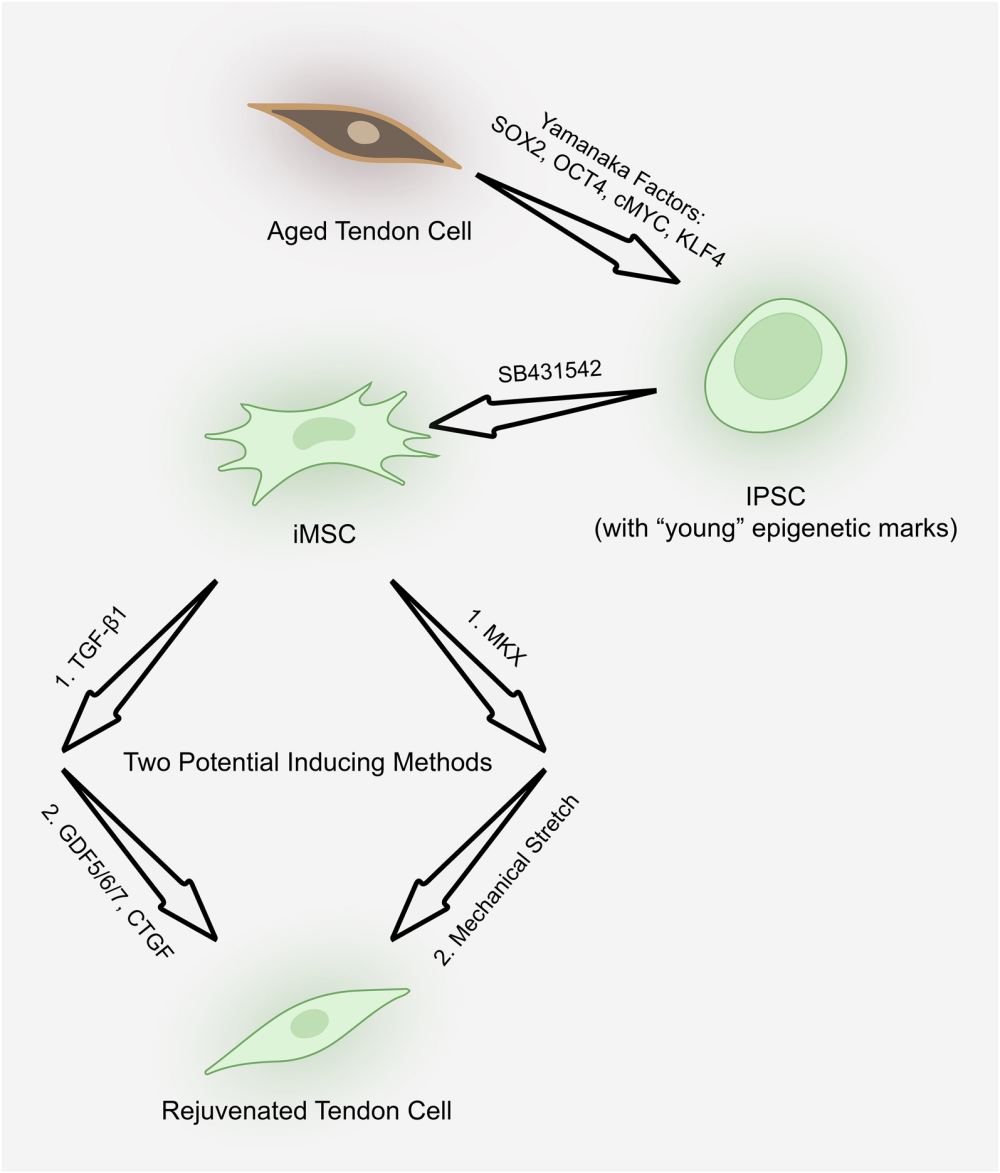
Proposed method to reprogram tendon cells from aged to rejuvenated. Aged tendon cell is possible to be reprogrammed to IPSC by Yamanaka factors and further induced to iMSC. There are two potential pathways for differentiation from iMSC to IPSC. IPSC, induced pluripotent stem cell. iMSC, IPSC-derived mesenchymal stem cell

Cells have undergone dramatic changes in their epigenome during ageing. A critical challenge is that aged tendon cells are not ideal to be used as donor cells for cell therapy. Here, we propose a reprogramming method to produce rejuvenated tendon cells based on the following information (Fig. 4). A recent attempt has produced rejuvenated MSCs from older people through the generation of IPSCs. IPSCs have been proven to have the ability to rejuvenate aged cells by transfection of four Yamanaka factors. These factors, SOX2, OCT4, cMYC and KLF4, can reprogram and rejuvenate the aged cells by acquiring “young” epigenetic marks [193]. Rejuvenated IPSCs can be further differentiated into IPSCs derived MSCs (iMSCs) by incubation with the transforming growth factor-β pathway inhibitor SB431542 [194]. Using this method to derive iMSCs from rejuvenated IPSCs, the iMSCs acquired young signature at their epigenome as well when compared with young MSCs and aged MSCs [195]. This is a huge step towards the generation of rejuvenated TDSCs from older people. Stepwise differentiation of MSCs into tendon cells was initiated by TGF-β1 to stimulate tenogenesis in BMSCs, by increasing transcription factor SCX significantly [196], and followed by supplementation of GDF 5/6/7/CTGF to induce and maintain full tenocyte-lineage commitment as subcutaneous injection of GDF 5/6/7 implants induced ectopic tendon-like structure in rat and CTGC promoted tenocyte differentiation [197]. This stepwise tenogenic differentiation from MSC can be completed in 10 days [196]. Further in vivo data of tendon repair by the iMSCs-derived tendon cells are required to standardise this stepwise differentiation. It remains unknown whether the MSCs differentiation into tenocytes is via TDSC as an intermediate status. Further investigation on the expression of differential genes between TDSCs and differentiated tenocytes at different steps should be carried out to determine this intermediate status. Additionally, the mechanism of converting iMSCs to TDSCs also remains to be determined. A recent study has shown that MKX combined with mechanical stretch could generate tendon-like tissue from IPSCs, which provides supporting evidence for converting iPSCs to tendon cells [198].

#### Hypoimmunogenic tendon cells derived from hypoimmunogenic IPSCs

Although ATI has been proven to be an efficient technique for tendon repair, it is still limited in its application as autologous fit tendon cells are limited. Unfit cell sources with specific mutations could be possibly modified by CRISPR/CAS9 in vitro [199]. However, this will not be practical if telomere segments are dramatically shortened and numerous mutations are accumulated in the genome during ageing or in some genetic diseases with genome instability [200, 201]. Allogenic tendon cell implantation may be the way to overcome this difficulty when the source of IPSCs is hypoimmunogenic. Moreover, establishment of allogenic tendon cells implantation can develop tendon-derived cell therapy into a more standardised treatment with consistent treatment efficacy. IPSCs can become hypoimmunogenic by knocking out of B2M, HLA subtypes or/and overexpression of CD47 [202, 203], as these strategies could affect the antigen presentation to T cells and trigger T cell response and thus lead to the surface-epitope masking. By directing hypoimmunogenic IPSCs differentiation into iMSCs, hypoimmunogenic tendon-derived cells could potentially be obtained via the aforementioned stepwise differentiation and could ultimately provide unlimited sources of tendon cells for multiple recipients for tendon repair in the future.

## Concluding remarks and future directions

Emerging tendon-derived cell therapies have offered significant and sustained clinical improvements in pain and functional outcomes as a result of tendinopathy. This review summarises two major challenges surrounding this therapy: (1) the effects of differentiation status of tendon-derived cells used in cell therapy, such as the feasibility for the treatment of enthesis, MTJ and midsubstance in tendons and (2) fitness of tendon-derived cells for tendon-derived cell therapy. Defining tendon cells subpopulations in enthesis, MTJ and midsubstance of tendons assists in addressing the first challenge. Cutting edge technologies such as single-cell transcriptome and spatial transcriptome are effective tools in identifying the cells in those three areas of tendons, although it is noted that transcriptome data are limited to midsubstance of tendons. The efficacy of cell therapy using tendon cells extracted from each site should then be systemically evaluated for each distinct injury site. The fitness of tendon-derived cells contributes to the success of tendon cell derived therapy. Factors affecting tendon cell fitness include ageing, physical and chemical exposure. Tendon-derived cells carrying defective gene mutations are considered less effective for tendon-derived cell therapy.

Both differentiation status and fitness of tendon cells must be considered in the implementation of tendon-derived cell therapy for specific circumstances. Mitochondrial transfer, microRNA, enzymes, recombinant proteins, physical stimulation and exosomes treatment have been shown to improve function of tendon cells. With this in mind, we propose a reprogramming protocol to produce rejuvenated tendon cells from aged cells utilising recent advances in gene editing technology. This allows for defective gene mutations to be corrected in vitro, and more importantly, hypoimmunogenic tendon-derived cells can be generated and used as a universal source for tendon-derived cell therapy for multiple recipients. Further development regarding the generation of hypoimmunogenic tendon-derived cells is an exciting concept to be further explored in the field of tendon-derived cell therapy.

## Data Availability

Not applicable.

## Abbreviations

MTJ: Myotendinous junction
TDSCs: Tendon-derived stem cells
ADLs: Activities of daily living
NSAIDs: Non-steroidal anti-inflammatory drugs
PRP: Platelet-rich plasma
GMP: Good manufacture practice
FDA: Food and drug administration
BMSC: Bone marrow-derived stem cell
ADSC: Adipose-derived stem cell
MSCs: Mesenchymal stem cells
OCT4: Octamer-binding transcription factor 4
SCX: Scleraxis
ATI: Autologous tenocyte implantation
ECM: Extracellular matrix
BMP2: Bone morphogenetic protein 2
SSEA-4: Stage-specific embryonic antigen-4
TPPP3: Tubulin polymerisation-promoting protein family member 3
+ve: Positive
−ve: Negative
SOX9: SCX/SRY-box transcription factor 9
GLI1: GLI family zinc finger 1
PM: Paraxial mesoderm
LPM: Lateral plate mesoderm
CNCC: Cranial neural crest cells
MKX: Mohawk homeobox
TNMD: Tenomodulin
ROBO: Roundabout
LRT: Leucine-rich tendon-specific protein
FAK: Focal adhesion kinase
GDFs: Growth/differentiation factors
CTGF: Connective tissue growth factor
CFTR: Cystic fibrosis transmembrane conductance regulator
EGR1: Early growth response 1
HO: Heterotopic ossification
DAMPs: Damage-associated molecular patterns
αSMA: Alpha smooth muscle actin
tHO: Heterotopic ossification occurs in trauma
bHO: Heterotopic ossification occurs in hyperactive BMP conditions
OSX: Osterix
CT: Computerised tomography
MRI: Magnetic resonance imaging
FAPs: Fibro/adipogenic progenitor cells
T-FAPs: Tendon fibro/adipogenic progenitor
SA-β-gal: Senescence-associated β-galactosidase
SPARC: Secreted protein acidic and rich in cysteine
ACL: Anterior cruciate ligament
RC: Rotator cuff
Botox: Botulinum toxin
mtDNA: Mitochondria DNA
PIN1: Peptidyl-prolyl cis-trans isomerase NIMA-interacting 1
FOXP1: Forkhead box P1
IPSCs: Induced pluripotent stem cells
iMSCs: Induced pluripotent stem cells-derived MSCs
HLA-DR: Human leukocyte antigen-DR isotype
TNC: Tenascin C
